# Identifying patient priority targets for improving a transitional care young adult rheumatology service: a group concept mapping evaluation

**DOI:** 10.1093/rap/rkaf118

**Published:** 2025-10-11

**Authors:** Alex Hughes, Gemma O’Callaghan, Gemma Bradley, Lesley Kay, Elizabeth Kidd, Katie L Hackett

**Affiliations:** School of Communities and Education, Northumbria University, Newcastle upon Tyne, UK; Rheumatology, Freeman Hospital, Newcastle upon Tyne Hospitals NHS Foundation Trust, Newcastle upon Tyne, UK; School of Communities and Education, Northumbria University, Newcastle upon Tyne, UK; Rheumatology, Freeman Hospital, Newcastle upon Tyne Hospitals NHS Foundation Trust, Newcastle upon Tyne, UK; National Clinical Director for Musculoskeletal Conditions, NHS England, London, UK; Rheumatology, Freeman Hospital, Newcastle upon Tyne Hospitals NHS Foundation Trust, Newcastle upon Tyne, UK; School of Communities and Education, Northumbria University, Newcastle upon Tyne, UK

**Keywords:** service evaluation, mixed methods, arthritis, multidisciplinary care, autoimmune diseases, patient communication

## Abstract

**Objectives:**

The study aimed to identify key priorities for improving a transitional adolescent and young adult rheumatology service through a comprehensive mixed methods service evaluation.

**Methods:**

We used a group concept mapping methodology. Patients (ages 16–25 years) attending an adolescent and young adult (AYA) rheumatology clinic completed idea generation, sorting and rating activities related to their experiences in the AYA service. Multidimensional scaling and hierarchical cluster analysis were applied to the sorted ideas to generate a concept map containing themed clusters of ideas. Rating data were analysed to identify themed clusters containing specific ideas to determine whether these were being successfully met and to identify specific targets for future service improvements.

**Results:**

A total of 48 patients participated in idea generation, 10 in sorting and 37 in rating activities. The concept map revealed six key themed clusters: Rheumatology Service Contact and Personal Information, Employment and Education Support and Advice, Mental Health and Well-being Support, Education and Advice About My Condition, General Clinic Improvements and Young Adult Specific Clinic Improvements. Specific improvement targets were identified within each of these themed clusters.

**Conclusion:**

This group concept mapping evaluation identified that the AYA rheumatology service was successfully meeting the needs of patients in many ways and helped to identify specific targets for future improvements. The resulting concept map provides a platform for use in partnership with patients to facilitate the co-design of future clinic improvements.

Key messages

- Young adult rheumatology patients require holistic support, including assistance with employment, health education and mental health.
- Improved access and usability of digital communication and self-management support would enhance rheumatology care for young adults.
- This comprehensive evaluation can be used with patients to facilitate future clinical care improvements.

## Introduction

Around one-sixth of the UK population experiences a rheumatic condition [1], and it is recommended that those with core inflammatory conditions should receive support from specialist rheumatology teams [2]. For those diagnosed in childhood, lifelong contact with rheumatology services is often required [3–5].

UK rheumatology departments are traditionally divided into paediatric and adult services [6]. However, adolescence and young adulthood present unique developmental challenges, highlighting the need for tailored transitional care [6, 7]. The Barbara Ansell National Network for Adolescent Rheumatology and guidance from the National Institute for Health and Care Excellence in the UK emphasise the importance of developmentally appropriate transitional pathways to support self-management during this critical period [8–10]. Despite these recommendations, a European study found dissatisfaction with transitional care [3]. Integrating patient-identified priorities into transitional services has the potential to enhance satisfaction and outcomes for young people with juvenile-onset rheumatic conditions [11].

In response, a National Health Service (NHS) rheumatology service in northeast England reconfigured its adolescent and young adult service to deliver holistic, multidisciplinary care for individuals ages 16–25 years, led by a named rheumatologist with a specialist nurse. A specific young adult outpatient clinic is run three times a month and patients from this clinic may also access the adult day unit for treatment, including infusions when needed. Adolescents transitioning from the NHS Trust’s paediatric service attend an initial joint clinic at the Great North Children’s Hospital, where both adult and paediatric rheumatologists see them. Transition to the adult clinic (at a different hospital site) is arranged with the young person’s agreement and additional joint appointments can be offered if needed. A comprehensive document outlining the adult service—including team photos, clinic location and contact details—is provided. The first adult appointment includes team introductions and a department and day unit tour. A paediatric rheumatology nurse specialist attends if indicated.

We aimed to evaluate the service and plan future patient-identified improvements by identifying service-users’ needs, prioritising their importance, assessing whether these needs were being met and identifying specific target areas for future service improvements.

## Methods

### Design, setting and sample

We used a group concept mapping (GCM) [12] mixed methods participatory design utilising several group processes (idea generation, sorting and rating activities) and multivariate statistical analysis (multidimensional scaling and hierarchical cluster analysis) to produce co-authored concept maps. This approach has been used in similar healthcare settings to design, evaluate and improve interventions and services [13, 14]. The concept maps visually represent how participants perceive and organise relationships between ideas on a topic [15], helping to identify target areas for service improvement [16].

Patients 16–25 years of age who accessed an adolescent and young adult (AYA) rheumatology service were invited to participate in one or more stages of this GCM evaluation over a 22-month period from January 2022 to October 2023. Participants were invited to complete paper surveys while attending the outpatient clinic or day-case unit or they received a postal survey. Anonymity was maintained at all stages, with only demographics (age, diagnosis, gender and length of time they had been accessing the AYA clinic) being collected, meaning it was not possible to determine which participants took part in more than one stage of the process.

### Data collection & analysis

#### Stage 1: Idea generation

This was conducted by a final-year occupational therapy student (A.H.). Participants completed a paper-based brainstorming exercise, responding to an incomplete focus prompt and providing a statement for each unique idea:

One specific way the adult rheumatology service can support me to do things I want and need to do in life is…

A.H., G.O. and K.L.H. conducted an interim analysis of statements as they were collected. Data collection continued until saturation was reached [17], with no new ideas emerging in response to the focus prompt. All statements were entered into a spreadsheet for stage 2.

#### Stage 2: Statement reduction

The statement reduction step was conducted qualitatively by two members of the research team (K.L.H., A.H.), consistent with standard GCM practice [12]. This approach prioritised preserving the original meaning and intent of participants’ contributions, ensuring unique and clear statements for subsequent sorting. Specifically, statements containing more than one idea were split into two separate statements and duplicate ideas were eliminated. Where necessary, statements were edited for readability. The final set of statements was uploaded to the GroupWisdom GCM web platform (Concept Systems, Ithaca, NY, USA), randomised and each statement was allocated a unique number.

#### Stage 3: Sorting

Each numbered statement was printed onto an individual card. Participants were asked to complete a sorting exercise in which they grouped statements into piles of similar meaning and assigned a name to each pile, representing its contents. As is typical in GCM studies, the sorting phase involved a smaller number of participants due to its cognitively demanding nature, particularly in clinical settings where participant burden must be minimised [18]. Participants were instructed to create more than one themed pile and not to make a ‘miscellaneous’ pile. They were then asked to document the name of each pile on a separate sheet of paper along with the corresponding numbers for the sorted statements.

#### Stage 4: Rating

Participants were asked to rate each of the 49 statements for importance and current success on a 1–5 Likert scale, with 1 being unimportant or need unsuccessfully met and 5 being very important or need successfully met.

#### Stage 5: Data analysis/interpretation

Sorting and rating data were analysed using GroupWisdom software. The sorting data were converted into a binary similarity summed square matrix and multidimensional scaling (MDS) was applied to produce a two-dimensional point map that depicts each numbered statement and the relationships between them. Statements frequently sorted together by service users are located nearer to each other on the map, whereas items that have been infrequently sorted together are located further away. A stress value was calculated, indicating the discrepancy between values within the similarity matrix and distances on the two-dimensional map, with a lower value indicating better goodness of fit [12]. The acceptable stress value range for GCM projects is 0.205–0.365 [19]. Hierarchical cluster analysis (Ward’s algorithm [20]) was next performed to group the numbered statements into conceptually themed, non-overlapping clusters. Maps containing as many as 15 clusters and as few as 4 were considered during two interpretation sessions by the authorship team. The software suggested cluster names based on names given to the sorted piles during the sorting exercise. A final cluster solution was agreed upon, with the cluster names determined following a third interpretation meeting involving two project team members (K.L.H., G.O.), three service users (one male and two females, ages 17–25 years) and one parent.

Importance and current success rating data were analysed at a cluster level to produce pattern matches where mean importance and current success scores were assigned to each cluster. Additionally, rating data were analysed at the individual statement level and a bivariate go-zone value plot was generated. Mean importance and success scores were used as a cut-off to divide the plots into four quadrants. A high-priority idea, which scores above the mean for current success, will fall within the top right quadrant of the go-zone and is identified as a high-importance/high-success statement. Statements with a rating above the mean for importance but below the mean cut-off for success are identified as improvement targets [13]. The lower left quadrant also includes statements of low success, but participants rate these as less important. Therefore, they may be considered secondary areas for future improvements.

### Ethical approval

The NHS Trust’s Research and Development Department assessed the project and determined that it was a service evaluation. It was registered as such (project 13430) and was therefore exempt from formal ethical approval by the Health Research Authority. A verbal explanation of the project was provided to all potential participants and participation was entirely voluntary. Patients were informed that declining to take part would not affect their clinical care.

## Results

### Participants

Fifty patients were invited to participate in the idea generation exercise, 10 were invited to sort and 41 were invited to rate. There were 48 participants in brainstorming, 10 in sorting and 37 in the rating activities. Initially, 107 statements were generated in response to the focus prompt. These were distilled into a final list of 49 unique statements for the sorting and rating exercises. Anonymity was maintained at all stages, and since only demographics were recorded (Supplementary Table S1), it was not possible to determine whether the same participants participated in more than one stage of the process. Participants presented with a range of rheumatic conditions, with most participants having been in the service for >2 years (Table 1).

**Table 1.** Demographics of participants at each stage of the GCM exercise.

Participants	Stage		
	Brainstorming activity	Sorting activity	Rating activity
Age, years, mean (s.d.)	20.98 (2.92)	20.20 (2.10)	21.65 (2.50)
Gender, *n*			
Female	31	6	27
Male	17	4	9
Non-binary	–	–	1
Diagnosis, *n*			
JIA	35	6	19
RA	4	4	7
Granulomatosis with polyangiitis	1	–	1
Systemic lupus erythematosus	1	–	3
Psoriatic arthritis	1	–	3
Axial spondylarthritis	1	–	–
Chronic recurrent multifocal osteomyelitis	1	–	–
Behcet’s disease	2	–	1
Mixed connective tissue disease	1	–	1
Orbital myositis	–	–	1
Uveitis	–	–	1
Unspecified	1	–	–
Time in adolescent/young adult service, *n*			
0–6 months	8	3	5
7–12 months	2	–	3
1–2 years	3	1	6
≥2 years	35	6	23
Total participants, *n*	48	10	37

### Concept maps, pattern matches and go-zone

**Figure 1.**
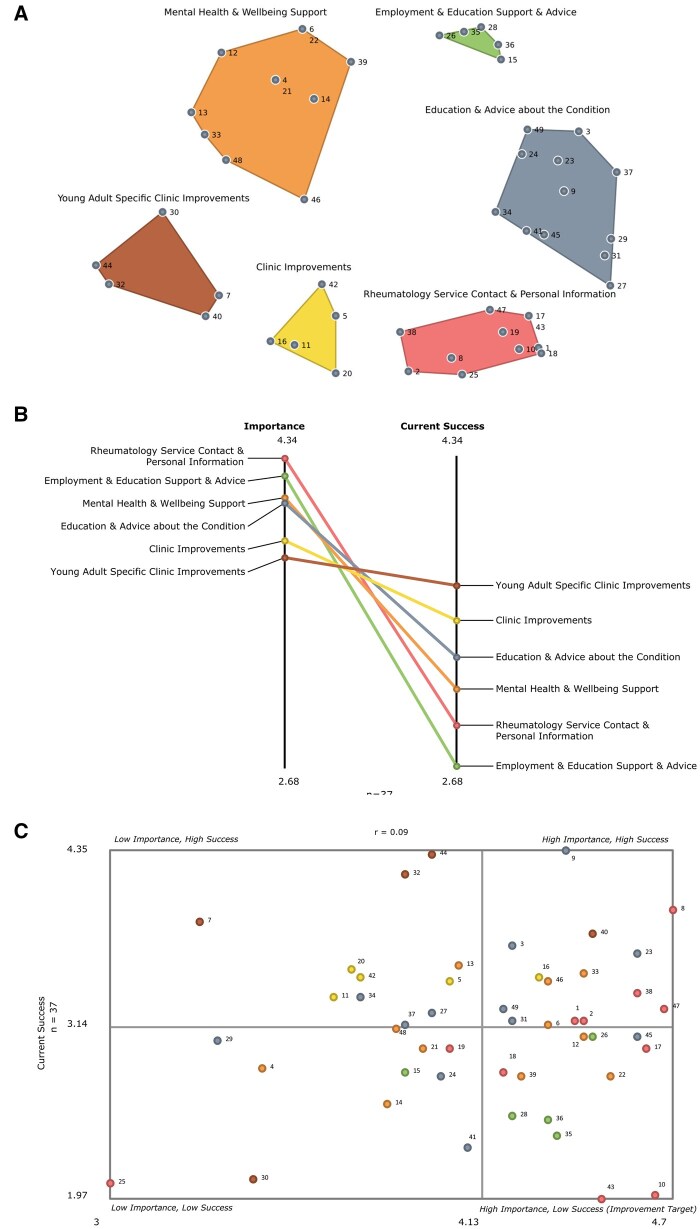
Concept maps, including a themed cluster map and corresponding numbered statements, a pattern match demonstrating importance and success ratings for each cluster and go-zone and identified target improvements among all statements. **(A)** Themed cluster map with corresponding numbered statements. **(B)** Pattern match with mean importance and success ratings for each cluster. **(C)** Go-zone containing numbered statements. The points for each numbered statement are the same colour as their representative cluster in the themed cluster map

Multidimensional scaling resulted in a point map with a stress value of 0.2738 following 16 iterations, demonstrating a good fit of the MDS point map to the original data [19]. A six-cluster solution (Fig. 1A) was agreed upon, which contained the following named clusters: Rheumatology Service Contact and Personal Information, Employment and Education Support and Advice, Mental Health and Well-being Support, Education and Advice About My Condition, General Clinic Improvements and Young Adult Specific Clinic Improvements. The smallest clusters (General Clinic Improvements, Young Adult Specific Clinic Improvements and Employment and Education Support and Advice) each contained 5 statements and the largest cluster (Education and Advice About My Condition) contained 12. The pattern match (Fig. 1B) is a visual representation of the mean importance and current success scores for each cluster. The go-zone shows categorised individual statements within priority and success categories within the plot (Fig. 1C). Statements falling within the upper right quadrant indicate a higher priority and greater success. In contrast, those in the lower right quadrant are likely targets for improvement, as they score above the mean importance score but below the mean average for current success.

Mean cluster scores and mean ratings for the statements within each cluster are presented in Table 2. The cluster Rheumatology Service Contact and Personal Information was deemed the most important (*M* = 4.34) and included 11 statements. Many statements focused on accessing their information within the service, e.g. #10 Creation of a patient-accessible platform to access blood results, appointments & prescriptions, #1 Access to medical history and #38 Having a named rheumatology professional who is accessible. Within this cluster, five important statements were successfully met and four targets for improvement were identified (Table 2).

**Table 2.** Mean importance and success ratings for the clusters and their corresponding statements

Statement number	Themed clusters and corresponding statements	Mean importance (1–5)	Mean current success (1–5)
	Cluster: Rheumatology Service Contact and Personal Information (11 statements)	**4.34**	**2.90**
8^a^	Text/e-mail reminders for appointments	4.70	3.95
47^a^	Having a helpline to contact for non-urgent medical advice	4.68	3.27
10^b^	Creation of a patient-accessible platform to access blood results, appointments and prescriptions	4.65	2.00
17^b^	Make it easier to get in contact with a member of the rheumatology medical team	4.62	3.00
38^a^	Having a named rheumatology professional who is accessible	4.59	3.38
2^a^	Make it easier to get an appointment	4.43	3.19
1^a^	Access to medical history	4.41	3.19
43^b^	Provide an e-mail address to ask non-urgent questions	4.49	1.97
18^b^	Access to blood results	4.19	2.84
19	Improved coordination between blood monitoring and clinic appointments	4.03	3.00
25	Development of app/text reminders to prompt exercises/movement	3.00	2.08
	Cluster: Employment and Education Support and Advice (5 statements)	**4.25**	**2.68**
26^b^	Support to access adjustments in school/college/university (e.g. rest breaks in exams)	4.46	3.08
35^b^	Advice for employers so they understand the needs of an employee with arthritis	4.35	2.41
36^b^	Liaison with school/college/university services to promote understanding of my condition	4.32	2.51
28^b^	Opportunity for rheumatology staff to discuss with employers my needs in the workplace/education	4.22	2.54
15	Employment support for me (e.g. managing work/life balance)	3.89	2.84
	Cluster: Mental Health and Well-being Support (11 statements)	**4.13**	**3.79**
22^b^	Mental health support for young people exposed to a hospital environment from a young age	4.51	2.81
12^b^	Provision of mental health support when diagnosed with a lifelong condition	4.43	3.08
33^a^	Regularly being asked about my mental health in rheumatology appointments	4.43	3.51
6^a^	Better signposting to other services for support with mental health	4.32	3.16
46^a^	Appointments that fit around work commitments	4.32	3.46
39^b^	Offering occupational therapy to everyone to support mental health and day-to-day life	4.24	2.81
13	Rheumatology professionals to ask questions about my day-to-day life/roles/routines to better understand me	4.05	3.57
21	Support to maintain participation in day-to-day activities such as socialising	3.95	3.00
48	Better introduction to the adult service rheumatology team to improve familiarity and reduce anxiety	3.86	3.14
14	Support in managing sleep	3.84	2.62
4	Support to maintain participation in sport activities	3.46	2.86
	Cluster: Education and Advice About My Condition (12 statements)	**4.10**	**3.27**
45^b^	Information about how best to reduce long term impacts of diagnosis	4.59	3.08
23^a^	Better explanation of diagnosis	4.59	3.65
9^a^	Understanding of how to take my medication and for how long	4.38	4.35
31^a^	Understanding specific things I can/can’t do when on my medication (e.g. drink alcohol, have piercings/tattoos etc.)	4.22	3.19
3^a^	Better explanations of treatments that are different in adult services from paediatric services (e.g. different medications used)	4.22	3.70
49^a^	Support to live a normal life while taking medication	4.19	3.27
41	Publish a handout that details different forms of support available within the rheumatology service (e.g. occupational therapy, physiotherapy, podiatry)	4.08	2.32
24	Understanding whether I am classed as disabled?	4.00	2.81
27	Access to information regarding the impact of COVID-19 on condition and on medication	3.97	3.24
37	Explanation of how the adult rheumatology service operates differently from the paediatric service to improve the transition between them	3.89	3.16
34	More information about what to expect as a day-case patient	3.76	3.35
29	Clearer advice on pregnancy planning	3.32	3.05
	Cluster: General Clinic Improvements (5 statements)	**3.90**	**3.46**
16^a^	Appointments that don’t interfere with education	4.30	3.49
5	Reduce waiting times within clinics	4.03	3.46
42	More regular appointments/reviews	3.76	3.49
20	Space in clinical areas for mobility aids	3.73	3.54
11	Longer appointments to discuss concerns in more detail	3.68	3.35
	Cluster: Young Adult Specific Clinic Improvements (5 statements)	**3.81**	**3.65**
40^a^	Seeing the same consultant/nurse each time I visit	4.46	3.78
44	Giving patient choice of having parents in appointment	3.97	4.32
32	Having the option not to inform patients’ parents about information disclosed in appointments	3.89	4.19
30	Being in an environment with other young people rather than older people to access peer support	3.43	2.11
7	Less formality in appointments	3.27	3.86

Statement numbers in left-hand column correspond to the numbers depicted in the concept map (Fig. 1A) and go-zone plots (Fig. 1C). Mean scores for each cluster are in bold.

a High importance/high success (upper right quadrant of the go-zone).

b High importance/low success (lower right quadrant of the go-zone and identified as an improvement target).

The Employment and Education Support and Advice cluster highlighted coordination between services and school/work settings, such as #28 Opportunity for rheumatology staff to discuss with employers my needs in the workplace/education and #36 Liaison with school/college/university services to promote understanding of my condition. Four statements within this cluster were identified as being targets for improvement.

Mental Health and Wellbeing Support included 11 statements, with some relating to specific mental health support, e.g. #12 Provision of mental health support when diagnosed with a life-long condition. This cluster also contained statements related to facilitating mental health through improving participation in important occupations, such as #21 Support to maintain participation in day-to-day activities such as socialising and #4 Support to maintain participation in sport activities. Three statements relating to mental health support were identified as improvement targets.

The Education and Advice About My Condition cluster includes statements relating to knowledge, understanding and empowerment when living with a rheumatic condition, including #31 Understanding specific things I can/can’t do when on my medication (e.g. drink alcohol, have piercings/tattoos etc), #29 Clearer advice on pregnancy planning and #49 Support to live a normal life while taking medication. Further statements relating to guidance and accessing support included #45 Information about how best to reduce long-term impacts of diagnosis and #49 Publish a handout that details different forms of support available within the rheumatology service, e.g. occupational therapy, physiotherapy, podiatry. One statement within this cluster was identified as a target for improvement.

The general ‘Clinic Improvements’ cluster was deemed conceptually distinct from the ‘Specific Young Adult Clinic Improvements’ cluster by service users during the third interpretation session. Service users perceived statements #11 Longer appointments to discuss concerns in more detail and #5 Reduce waiting times within clinics as reflecting desired changes to clinics in general, regardless of age or life stage, whereas statements such as #30 Being in an environment with other young people rather than older people to access peer support and #32 Having the option not to inform patients’ parents about information disclosed in an appointment related to the unique needs of young adults transitioning from paediatric to adult services. Four improvement targets were identified within the general ‘Clinic Improvements’ cluster. All statements within the ‘Specific Young Adult Clinic Improvements’ cluster were rated as successful, with no statements falling into the targets for improvement quadrant in the go-zone (Fig. 1C).

## Discussion

We successfully engaged young adult patients accessing an AYA rheumatology service in a local evaluation and planning project using a novel participatory process to identify how the service is meeting the needs of users and to create an actionable list of priority quality improvement targets to improve the delivery of care to patients who have recently transitioned from paediatric to adult rheumatology care.

The themes emerging from our concept mapping strongly reflect priorities identified in the rheumatology transition literature. EULAR/Paediatric Rheumatology European Society (PReS) consensus standards [7] emphasise early, developmentally appropriate preparation, coordinated care, access to information and the support of a transition coordinator. These are echoed in our Rheumatology Service Contact and Personal Information and Mental Health and Well-being Support clusters. A previous qualitative systematic review [11] identified persistent concerns around emotional readiness, autonomy and relational continuity, which align with participant priorities such as Statement 38 (a named professional), Statements 12, 22 and 33 (mental health support) and Statements 10, 17 and 43 (communication access). McDonagh and Farre’s review [10] also identified the importance of starting transition early with personalised and digital support pathways. This is captured in our Access to Medical Information cluster. While previous research has largely documented these themes qualitatively, our structured, patient-led framework provides an actionable tool to inform service development and benchmarking.

The pattern match (Fig. 1B) highlights the disparity between the reported mean importance and success of each cluster. This is not uncommon in GCM projects, as participants often use the importance scale to express their values and a scale such as current success to highlight gaps or deficiencies in capacity [21]. It could be suggested that most of the 48 participant-generated statements within the six clusters should be regarded as potential improvement targets, as all scored ≥3 for importance [22]. However, for this article we followed the approach used by another GCM clinical evaluation [16] and focused primarily on statements within the bottom right quadrant of the go-zone (above mean importance/below mean current success) and identified these as primary targets for improvement.

The cluster with the greatest mean importance and the second-lowest mean success rating is Rheumatology Service Contact and Personal Information, indicating that this cluster presents significant room for development. In particular, #10: Creation of a patient-accessible platform to access blood results, appointments and prescriptions requires an appropriate information technology system designed around the needs of patients. The NHS app was launched in 2019 to be a trustworthy and branded smartphone app for patients to find their health information and access NHS services [23], but the app has faced issues with usability [24]. Indeed, our evaluation suggests that patients may not be regularly accessing it or the app is not working seamlessly across primary and secondary care boundaries. Patients with long-term conditions need access to this information to support their self-management, but access to this information seems to be a persistent problem across both primary and secondary care in the UK [25]. As the NHS app is improved, all patients should soon be able to view their appointments and access their blood results. However, further exploration with our patients may be required to identify any barriers in this area. Other identified targets for improvement within this cluster included #17 Make it easier to get in contact with a member of the rheumatology medical team and #14 Provide an email address to ask non-urgent questions. Such statements suggest that, despite a generic rheumatology telephone helpline being available, which works well (#47), some young adults may prefer different forms of communication to access support. The use of technology, such as messaging, is often favoured by young adults with long-term conditions as a primary means of routine communication and interaction with their healthcare team due to its convenience and accessibility [26, 27]. Text/email reminders about appointments (#8) were identified as being both an important and successful statement within this cluster, and such technological reminders are known to increase attendance at outpatient clinics [28], and our results indicated that service users appreciated them.

The cluster with the second-greatest mean importance and the lowest mean success score is Employment and Education Support and Advice. Four statements belonging to this cluster fell within the improvement target quadrant of the go-zone, indicating that specific employment and education support is needed for this patient group. Young adults with rheumatic diseases face unique challenges in education and employment compared with older adults, necessitating tailored support strategies, which should form part of their holistic care [7]. The transition from education to the workforce is particularly critical, as individuals often must balance disease management with education, entering the workforce and career development. Gignac *et al*. [29] found that perceived social support with education and employment significantly influenced employment participation among young adults with rheumatic diseases, underscoring the importance of addressing these factors during this transitional period. In 2024, the UK government issued guidance to implement a rollout of integrating employment advisors within musculoskeletal care pathways in England [30]. As the proof-of-concept first stage progresses to the second, wider rollout stage in England during 2025, it will be crucial that young people with rheumatic diseases are made aware of the support now being provided and how to access it.

Young people identified a need for robust mental health support, and our findings support the need to integrate this support into our AYA rheumatology service. Children and young people diagnosed with a rheumatic disease face unique and significant psychological challenges [31], and early and sustained interactions with healthcare systems can lead to increased anxiety, feelings of isolation and difficulties in transitioning to independent adult care [6, 7]. Participants identified occupational therapy as supporting mental health and day-to-day life challenges. Therefore, integrating occupational therapy within young adult rheumatology services may help address the intersection of physical and psychological challenges experienced by this group. We have integrated occupational therapy within the AYA clinic following this evaluation.

Silverthorne *et al.* [32] explored with rheumatology health professionals the optimum ways of supporting rheumatology patients with psychological distress. One way was to embed the support within the team, including through occupational therapy. However, without dedicated roles, expertise and time, rheumatology health professionals may feel inadequately equipped to provide this support [32], and it would need to be appropriately resourced. The EULAR/PReS standards and recommendations for rheumatology transitional care acknowledge the importance of holistic patient care and specify that staff should be equipped with the skills and knowledge to address emotional and mental health issues [7]. People with RA are more susceptible to depression than the general population, as chronic inflammation disrupts the body’s physiological responses to stress, including the ability to cope, which effectively leads to depression and ultimately poorer long-term outcomes for patients experiencing higher levels of stress [33]. As such, the UK National Institute for Health and Care Excellence guidance on RA in adults recommends that psychological interventions such as cognitive coping skills should be made available to support people in living with their condition [34]. A recent study demonstrated that younger rheumatology patients experienced more stress than older patients during the COVID-19 pandemic [35]. The pandemic may also be negatively contributing to young adult patients’ mental health and well-being, which requires consideration by care teams.

One further improvement target, #45 Information about how best to reduce long term impacts of diagnosis, is located within the Education and Advice About My Condition cluster. The EULAR/PReS recommendations additionally highlight the importance of supporting patients with self-management, shared decision-making and promoting a healthy lifestyle [7]. Again, an equipped and resourced multidisciplinary care team is paramount to offer this support.

In this evaluation, we identified potential primary improvement targets in the lower right quadrant of the go-zone and are focusing our attention on these as the service is developed; other statements from the remaining quadrants may also be considered as potential secondary improvement targets. One example of this was statement #30 Being in an environment with other young people rather than older people to access peer support, which belongs to the Young Adult Specific Clinic Improvements cluster. Respondents regarded this statement as both less important and less successful. Consequently, it was not identified as a primary target for improvement. However, during the third data interpretation with service users, a service user pointed out that educational and social events specifically for people accessing the young adult clinic would be helpful, as this would facilitate introductions to others going through similar experiences. Therefore, following our initial focus on the priority targets within the improvement targets quadrant of the go-zone, the team intends to work with service users to address additional easily actionable secondary targets in the remaining quadrants, particularly those located near the mean cut-off boundaries.

To audit implemented service improvements, the success rating questionnaire will be repeated. Results will be compared with baseline data at both the statement and cluster levels to assess improvements in patient satisfaction.

### Strengths and limitations

Although this was an exploratory, single-centre evaluation and planning project within a single NHS trust in northeast England, we observed strong patient engagement within our transitional care young adult rheumatology service. As a result, our sample size falls within the acceptable range for GCM projects [12, 20]. However, we relied on a convenience sample of participants who were attending the clinic. Subsequently, we may have missed some crucial perspectives from those who were less engaged with the service. This GCM project was undertaken within a single clinical service, therefore the findings are not automatically generalisable beyond our host organisation. However, other transitional care young adult rheumatology services may use the generated statements to evaluate their services. While the sorting stage in this evaluation involved a smaller sample size (*n* = 10), which is at the lower end of typical GCM recommendations, the resulting stress value (0.2738) demonstrates good representational validity [20]. Although the participants who took part in sorting had a diagnosis of JIA or RA, including a more diagnostically diverse sample may have provided additional insights into condition-specific needs. Furthermore, while individuals with rarer rheumatic diagnoses contributed during the brainstorming and rating phases, they were not represented in the sorting phase. As a result, their perspectives may have been underrepresented in how statements were grouped into clusters, even though their ideas were fully incorporated into the final concept map. However, our aim with this evaluation was to identify patient priorities for age-appropriate, developmentally tailored transitional care. We consider this to be an area that transcends specific disease pathways. Future investigations could build on this work by exploring diagnosis-specific needs of patients accessing transitional care services.

## Conclusion

We conducted a single-centre mixed methods service evaluation of an AYA rheumatology service in the northeast of England. Our findings indicate that, while the clinic effectively met the needs of patients in many ways, specific improvement targets were identified across six key themes. High-priority improvement targets included addressing patient needs in areas such as employment and education support, mental health and well-being and technology to facilitate easy access to appointments, blood results and prescriptions, with options for non-urgent support via text and e-mail. A multidisciplinary service with suitably trained staff is required to deliver such developmentally appropriate, holistic transitional care.

## Supplementary Material

rkaf118_Supplementary_Data

## Data Availability

The data underlying this article will be shared on reasonable request to the corresponding author.
