# Supplementary material for: Identifying patient priority targets for improving a transitional care young adult rheumatology service: a group concept mapping evaluation
Source: Rheumatol Adv Pract. 2025 Oct 11;9(4):rkaf118. doi: 10.1093/rap/rkaf118 (PMC12597889; doi:10.1093/rap/rkaf118)
Supplement: rkaf118_Supplementary_Data [file rkaf118_supplementary_data.docx]

**Supplementary Table S1: Demographics of participants for each stage of the GCM exercise**

| **Participants** | **Stage** | | |
| --- | --- | --- | --- |
|  | **Brainstorming** | **Sorting** | **Rating** |
| **Age, mean (SD)** | 21.0 (2.39) | 20.2 (2.10) | 21.6 (2.50) |
| **Gender:**  Female | 31 | 6 | 27 |
| Male  Non-binary | 17  - | 4  - | 9  1 |
| **Diagnosis:** |  |  |  |
| Juvenile Idiopathic Arthritis  Rheumatoid Arthritis  Granulomatosis with  Polyangiitis  Systemic lupus erythematosus  Psoriatic Arthritis  Axial Spondylitis  Chronic Recurrent  Multifocal Osteomyelitis  Behcet’s Disease  Mixed Connective Tissue Disease  Orbital Myositis  Uveitis  Missing data | 35  4  1  1  1  1  1  2  1  -  -  1 | 6  4  -  -  -  -  -  -  -  -  -  - | 19  7  1  3  3  -  -  1  1  1  1  - |
| **Time in adolescent/young adult**  **service:**  0-6 months  7-12 months  1-2 years  2+ years | 9  2  3  34 | 3  -  1  6 | 5  3  6  23 |
| **Total participants** | 48 | 10 | 37 |
